# Statins for the Treatment of Pulmonary Hypertension in Patients with Chronic Obstructive Pulmonary Disease

**DOI:** 10.3389/fphar.2020.613761

**Published:** 2021-01-08

**Authors:** Chung-Yu Chen, Wen-Ting Wu, Ya-Ling Wang, Kuang-Ming Liao

**Affiliations:** ^1^ Master Program in Clinical Pharmacy, School of Pharmacy, Kaohsiung Medical University, Kaohsiung, Taiwan; ^2^ Department of Pharmacy, Kaohsiung Medical University Hospital, Kaohsiung, Taiwan; ^3^ Department of Medical Research, Kaohsiung Medical University Hospital, Kaohsiung, Taiwan; ^4^ Department of Pharmacy, Wan Fang Hospital, Taipei Medical University, Taipei, Taiwan; ^5^ Department of Internal Medicine, Chi Mei Medical Center, Tainan, Taiwan

**Keywords:** statin (HMG-CoA reductase inhibitor), pulmonary hyperetnsion, chronic obstructive pulmonary disease, mortality, outcome

## Abstract

**Background:** Patients with chronic obstructive pulmonary disease (COPD) are at risk for pulmonary hypertension (PH). The aim of our study was to investigate the benefit of statins for PH in patients with COPD.

**Methods:** The study enrolled 23 million individuals from Taiwan’s population database from January 1, 2002, to December 31, 2017. COPD patients who met the inclusion criteria were enrolled, and patients with lung cancer, less than one year of observation, specific drug therapy for PH and lung transplantation were excluded.

**Results:** A total of 643,131 COPD patients were included in the study, and only 12,308 patients developed PH during follow-up. Based on the inclusion and exclusion criteria, 8,577 PH patients were included in the cohort of patients with PH related to COPD for analysis. According to the definition of statin exposure, the final study population had 1,487 statin users and 7,090 statin non-users. The statin user group had a lower mortality related to PH than the non-user group (3.87 vs. 5.55 per 100 person-years, *p* < 0.001). The mortality rate for PH in the multivariate analysis (aHR = 0.78, 95% CI = 0.62–0.98, *p* = 0.046) was significantly lower for statin users than for non-users.

**Conclusion:** Statins seem to benefit patients with PH and COPD.

## Introduction

Pulmonary hypertension (PH) is defined as a mean resting pulmonary arterial pressure exceeding 25 mm·Hg, as evaluated by right heart catheterization. In the sixth World Symposium on PH (WSPH) update from 2018, PH is divided into five groups according to clinical presentation, hemodynamic characteristics, pathophysiology, and therapeutic strategy (Simonneau et al., 2019). Patients with PH due to obstructive lung disease [e.g., chronic obstructive pulmonary disease (COPD)] or restrictive lung disease) are classified into group 3. Among older patients above the age of 65 years, group-2 PH is the most frequent diagnosis (17%), followed by group-3 PH (14%) (Cottin et al., 2005; Hurdman et al., 2013; Solidoro et al., 2015). In a Taiwan epidemiological survey, PH related to COPD was the most common form of PH (Chang et al., 2016).

Current medical treatments for PH, including supportive therapy and new specific drugs in PH related to COPD, are lacking, and the efficacy is still uncertain; only long-term oxygen therapy can improve symptoms.

Our previous study showed that the statin user group had a lower incidence rate of PH than the nonuser group (1.43 vs. 1.97 per 1,000 person-years) (Wu and Chen, 2020).

Therefore, we further performed a nationwide, population-based, retrospective cohort study to explore the association between statins and mortality among patients with COPD and PH.

## Materials and Methods

### Study Population

The Taiwan National health insurance program, which covers 99.6% of the 23 million people in Taiwan’s population and 93% of hospitals, clinics, and pharmacies, created one of the largest and most comprehensive medical population databases in the world. We used the full population database and multiple causes of death data from the National health insurance program. The study was conducted at the Center of Health and Welfare Data Science Centers. The medical record of the full population database was used from January 1, 2002, to December 31, 2017.

To assess whether the use of statins before PH diagnosis affects the study outcome, the study population was divided into statin users, defined as statin exposure within one year, and statin non-users, defined as no statin prescription within one year. This study was approved by the IRB of Kaohsiung Medical University Chung-Ho Memorial Hospital (KMUHIRB-EXEMPT(I)-20190032).

### Inclusion Criteria

The inclusion criteria were 1) age between 40 and 90 years, 2) newly diagnosed COPD between January 1, 2002, and December 31, 2015 (according to International Classification of Diseases, Ninth Revision, Clinical Modification codes 490, 491, 492, and 496), 3) more than one inpatient diagnosis or more than two consecutive outpatient diagnoses of COPD within one year after the primary COPD diagnosis date, and 4) treatment with COPD medications indicated in outpatient claims for more than 28 days (COPD medication: long-acting beta 2 agonist, long-acting muscarinic antagonist, short-acting beta 2 agonist combined with oral methylxanthines or short-acting muscarinic antagonist combined with oral methylxanthines) (Wang et al., 2018), and 5) more than one inpatient diagnosis or emergency room admission for PH or more than two consecutive outpatient diagnoses of PH within one year. The date of the first diagnosis of PH was defined as the index date.

### Exclusion Criteria

The exclusion criteria were 1) COPD with lung cancer, 2) COPD with other etiologies of PH, including human immunodeficiency virus, sleep apnea, pulmonary embolism, connective tissue disease, congenital heart defect, or portal hypertension, 4) death within 28 days after COPD diagnosis, 5) specific drug therapy for PH treatment (e.g., sildenafil, riociguat) before COPD diagnosis, 6) death within 1 year after the first PH diagnosis date, 7) lung transplantation within 1 year after the first PH diagnosis, of 8) a less than one-year observation time in the database.

### Immortal Time Bias

To resolve the immortal time bias, we set a one-year confirmation period (Levesque et al., 2010). Patients who needed to start using statins and who had at least one prescription for statins within one year after the COPD diagnosis were defined as statin users. Conversely, patients who did not receive a prescription for statins within one year after the COPD diagnosis were defined as statin non-users.

### Baseline Characteristics

Baseline characteristics included age, age group, sex, urbanization level, insurance premium, comorbidities, and co-medication. Comorbidities and co-medication were confirmed by medical records in the year following the PH diagnosis (i.e., one year).

To adjust for other confounders that could influence the outcome, we listed the PH treatment [e.g., oxygen therapy, digoxin, calcium channel blocker (CCB), warfarin, endothelin receptor antagonists, phosphodiesterase type 5 inhibitors, prostacyclin analogs] in the baseline characteristics.

### Severity of Pulmonary Hypertension

The severity of PH is usually expressed by the WHO functional class, mean pulmonary artery pressure, or exercise capacity test, such as the six-minute walk distance. However, we could not collect those data from our database. This study used COPD severity within one year after the PH diagnosis, with oxygen therapy and heart failure (HF) used to represent PH severity. In the clinic, if patients have more severe COPD with concomitant HF, they are considered to have more severe PH. These three factors were included in the calculation of the propensity score to identify patients with the same PH severity.

### Propensity-Score Matching

We used propensity-score matching to reduce selection bias. This matching method calculated a propensity score for each patient by means of dependent variables. If patients had the same propensity score, they had equal probability of developing events that we wanted to observe. The baseline characteristics of the original cohort were used to perform propensity-score matching by multivariate logistic regression. We adopted 1:1 matching to generate the statin user group and statin non-user group after follow-up.

### Primary Outcome

The primary outcome was PH-related mortality, which was a composite outcome that included deaths caused by PH, deaths caused by HF, and deaths caused by respiratory exacerbation (RE). Deaths were identified using the “Multiple Cause of Death Data.” The “Multiple Cause of Death Data” contains patients’ dates of death, hospitals of death, and causes of death.

### Secondary Outcome

The all-cause mortality, deaths caused by PH, deaths caused by HF, and deaths caused by RE were secondary outcomes. The cause of death was identified by International Classification of Diseases, Ten Revision, Clinical Modification code according to the “Multiple Cause 1 to 20 (MC1-MC20)” columns from Multiple Cause of Death Data.

### Follow-Up Time

We set a five-year observation duration to reduce the influence of switching to different statins or refusal to take statins. The follow-up period started from one year after the index date to five years after the index date. The observation time ended at death or censor occurrence. The patients in both groups who were alive or underwent lung transplantation at the end of the observation period were censored.

### Subgroup Analysis

We conducted a subgroup analysis to assess whether the association variables could affect the effectiveness of statins. Only the primary outcomes were included in the subgroup analysis.

#### Different Kinds of Statins

We grouped patients treated with seven kinds of statins according into the group of most commonly used statins. The seven statins were simvastatin, lovastatin, pravastatin, fluvastatin, atorvastatin, rosuvastatin, and pitavastatin.

#### Cumulative Defined Daily Dose

The cDDD was the total statin exposure over the whole follow-up period. The cDDD was calculated over the entire five-year observation duration. The cDDD was divided into six levels in the user group compared to the non-user group: non-user, cDDD <28, cDDD ≥28 but <90, cDDD ≥90 but <180, cDDD ≥180 but <365, cDDD ≥365 but <730, and cDDD ≥730.

#### Duration of Statin Use (years)

The duration of statin use was calculated by year and divided into six levels in the user group compared to the non-user group: non-user, <0.5 year, ≥0.5 year but <1 year, ≥1 year but <2 years, ≥2 years but <3 years, and ≥3 years.

#### Intensity of Statin Use (Cumulative Defined Daily Dose/Month)

The intensity of statin use was calculated by dividing cDDD by the duration of statin use over the whole five-year observation period. Then, statin use was divided into four categories to determine whether the intensity could affect the protective effect of statins: non-user, intensity <10, intensity ≥10 ≤ but <20, intensity ≥20.

### Sensitivity Analysis

#### Different Observation Durations

The observation times for the sensitivity analysis were 1 year, 3, 5, 7, 9 years, and the end of the database to detect the effects of different observation durations.

### Statistical Analysis

For baseline characteristics, continuous variables are presented as the means (standard deviations), and categorical variables are presented as percentages. Continuous variables were analyzed by Student’s t-test or analysis of variance, and categorical variables were analyzed by Fisher’s exact test or chi-square tests if numbers were less than 30.

The mortality of PH was assessed using a time-dependent Cox proportional hazard model to estimate the hazard ratio between the two groups. The multivariate model was adjusted for demographic characteristics, sex, comorbidity, co-medication, and COPD severity. Because of potential interactions and collinearity between characteristics, stepwise selection was performed to select the important factors to construct the regression model. All the above analyses were performed using SAS 9.4. software. The statistical significance was determined with two-tailed analyses and α = 0.05.

## Results

### Study Population

**FIGURE 1:**
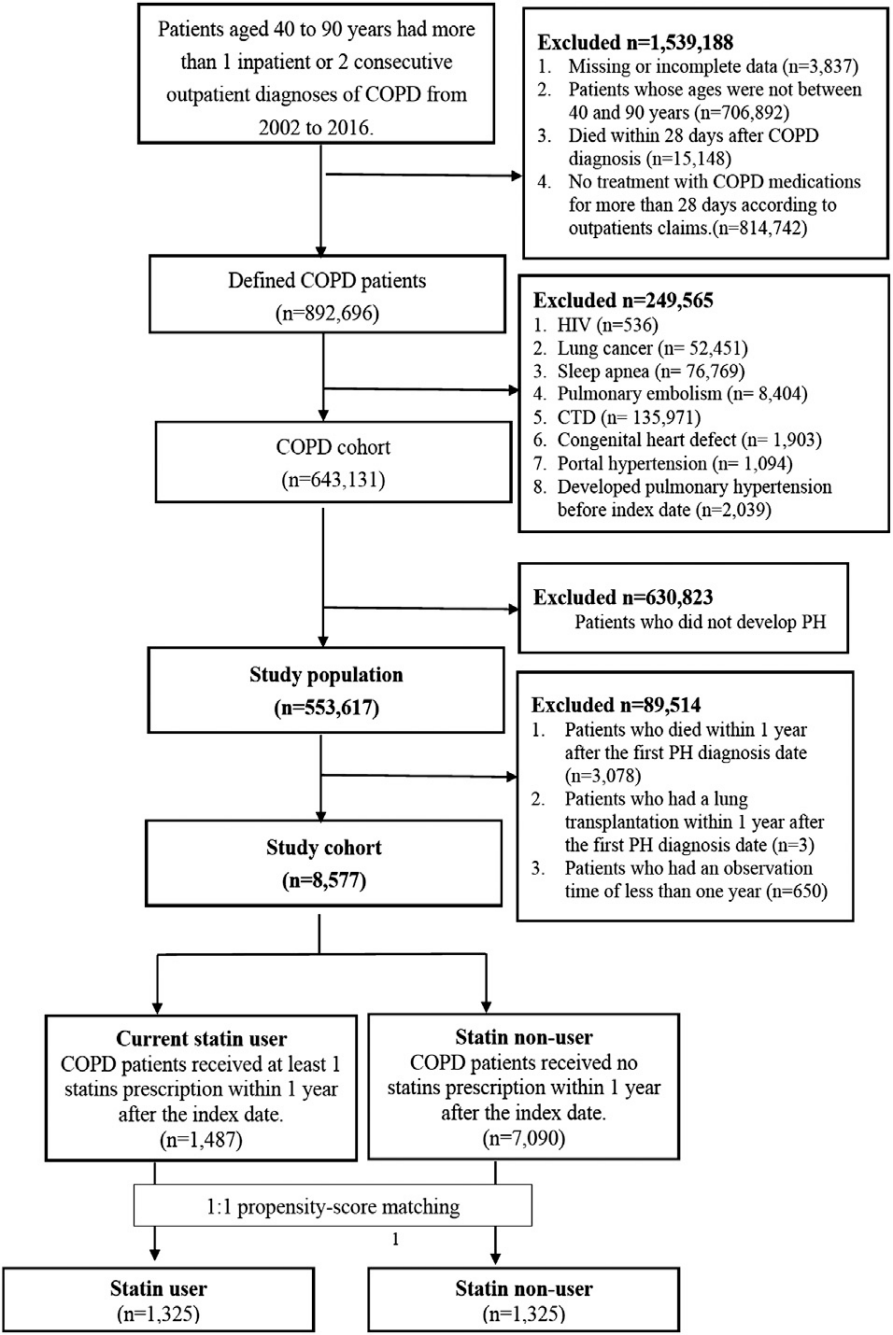
Result of flow chart in the study.

A total of 643,131 COPD patients were included in the study, and only 12,308 patients developed PH during follow-up. Based on the inclusion and exclusion criteria, 8,577 PH patients were included in the PH related to the COPD cohort for analysis. According to the definition of statin exposure, the final study population had 1,487 statin users and 7,090 statin non-users. Figure 1 showed the study flow chart.

### Baseline Characteristics

The PH cohort contained 1,487 statin users and 7,090 non-users. The mean age of the patients in the user group (72.52 years) was lower than that of the non-user group (74.10 years, *p* < 0.001). The age distributions were similar between groups, with most patients over 60 years old and the majority being male. The insurance premium showed the same distribution, with more patients paying below 22,800 Taiwan Dollars and a significant difference between the two groups (*p* = <0.001).

Because of the indications for statins, patients with statins had a significantly higher prevalence of dyslipidemia (*p* < 0.001), coronary artery disease (*p* < 0.001), and ischemic stroke (*p* < 0.001). The proportions of most comorbidities were significantly higher in the user group than in the non-user group. There was no significant difference in chronic liver disease, arrhythmia, interstitial pulmonary diseases, asthma, malignancy, hemorrhagic stroke or left ventricular hypertrophy between groups.

Co-medication, except with digoxin, had the same trend for comorbidity. More patients in the non-user group used digoxin (18.16% vs. 21.28%, *p* = 0.007), In addition, there were no significant differences in oxygen therapy or specific drug therapy between groups. Regarding medication for COPD, more than half of the patients used methylxanthines. Regarding the severity of COPD, there were significant differences in both severe and moderate exacerbations (*p* < 0.001) between the two groups. The distributions of severe and moderate exacerbations showed that most patients had no exacerbation in the firth year after COPD was diagnosed, and the second highest number of patients had over two exacerbations. The detailed baseline characteristics of the PH patients before matching are presented in Table 1.

**TABLE 1:** Baseline characteristics of PH patients before and after matching, stratified according to statin use.

Characteristics n (%)	User N = 1,487	Non-user N = 7,090	*p*-value	Matching User N = 1,325	Matching Non-user N = 1,325	*p*-value
Propensity score (SD)						
No matching				0.77 (0.12)	0.84 (0.10)	<0.001
Matching				0.66 (0.04)	0.66 (0.04)	1.000
Age group						
Mean (SD)	72.52 (10.43)	74.10 (10.73)	<0.001	72.64 (10.44)	72.67 (10.46)	0.935
40 ≤ age <50 years	29 (1.95)	176 (2.48)	<0.001	25 (1.89)	25 (1.89)	1.000
50 ≤ age <60 years	158 (10.63)	610 (8.60)		140 (10.57)	140 (10.57)	
60 ≤ age <70 years	338 (22.73)	1,313 (18.52)		303 (22.87)	303 (22.87)	
70 ≤ age <80 years	554 (37.26)	2,474 (34.89)		484 (36.53)	484 (36.53)	
age ≥80 years	408 (27.44)	2,517 (35.50)		373 (28.15)	373 (28.15)	
Male	826 (55.55)	4,527 (63.85)	<0.001	753 (56.83)	753 (56.83)	1.000
Insurance premium (TWD)						
≤22,800 TWD	923 (62.07)	5,066 (71.45)	<0.001	826 (62.34)	963 (70.64)	<0.001
>22,800 TWD	564 (37.93)	2024 (28.55)		499 (37.66)	389 (29.26)	
Urbanization level						
Urban	720 (48.42)	3,131 (44.16)	0.011	644 (48.60)	597 (45.06)	0.1868
Suburban	594 (39.95)	3,046 (42.96)		528 (39.85)	563 (42.49)	
Rural	173 (11.63)	913 (12.88)		153 (11.55)	165 (12.45)	
Comorbidity						
Dyslipidemia	780 (52.45)	352 (4.96)	<0.001	697 (52.60)	93 (7.02)	<0.001
Hypertension	1,109 (74.58)	3,946 (55.66)	<0.001	947 (71.47)	944 (71.25)	0.897
Diabetes mellitus	692 (46.54)	1,459 (20.58)	<0.001	530 (40.00)	528 (39.85)	0.937
Obesity	9 (0.61)	18 (0.25)	0.028	8 (0.60)	5 (0.38)	0.404
Chronic kidney disease	231 (15.53)	504 (7.11)	0.026	193 (14.57)	115 (8.68)	<0.001
Chronic liver disease	125 (8.41)	707 (9.97)	0.064	109 (8.23)	154 (11.62)	0.004
Arrhythmia	432 (29.05)	2,147 (30.28)	0.347	393 (29.66)	421 (31.77)	0.238
Interstitial pulmonary diseases	34 (2.29)	230 (3.24)	0.052	33 (2.49)	45 (3.40)	0.168
Asthma	902 (60.66)	4,284 (60.42)	0.866	797 (60.15)	792 (59.77)	0.843
Malignancy	183 (12.31)	893 (12.60)	0.760	158 (11.92)	166 (12.53)	0.635
ASCVD						
Coronary artery disease	746 (50.17)	2,112 (29.79)	<0.001	669 (50.49)	451 (34.04)	<0.001
Peripheral vascular disease	84 (5.65)	269 (3.79)	0.001	76 (5.74)	68 (5.13)	0.493
Ischemic stroke/TIA	246 (16.54)	820 (11.57)	<0.001	220 (16.60)	168 (12.68)	0.004
Hemorrhagic stroke	17 (1.14)	99 (1.40)	0.442	15 (1.13)	16 (1.21)	0.857
Heart failure	792 (53.26)	3,258 (45.95)	<0.001	719 (54.26)	710 (53.58)	0.726
Left ventricular hypertrophy	38 (2.56)	193 (2.72)	0.718	37 (2.79)	40 (3.02)	0.729
Co-medication						
Digoxin	270 (18.16)	1,509 (21.28)	0.007	247 (18.64)	304 (22.94)	0.006
Oral anticoagulant agents	219 (14.73)	744 (10.49)	<0.001	194 (14.64)	163 (12.30)	0.078
Oral antiplatelet agents	990 (66.58)	2,715 (38.29)	<0.001	889 (67.09)	578 (43.62)	<0.001
Diuretics	1,011 (67.99)	4,253 (59.99)	<0.001	897 (67.70)	870 (65.66)	0.266
CCB	874 (58.78)	3,371 (47.55)	<0.001	770 (58.11)	739 (55.77)	0.224
ACEI/ACB	1,015 (68.26)	3,165 (44.64)	<0.001	889 (67.09)	747 (56.38)	<0.001
Beta blocker	653 (43.91)	1707 (24.08)	<0.001	574 (43.32)	401 (30.26)	<0.001
Metformin	395 (26.56)	739 (10.42)	<0.001	314 (23.70)	247 (18.64)	0.001
Fibrates	78 (5.25)	109 (1.54)	<0.001	65 (4.91)	35 (2.64)	0.002
Oxygen therapy	252 (16.95)	1,273 (17.95)	0.355	14 (1.06)	3 (0.23)	0.007
Specific drug therapy	9 (0.61)	33 (0.46)	0.486	234 (17.66)	240 (18.11)	0.761
Medication for COPD						
LABA	64 (4.30)	345 (4.87)	0.355	60 (4.53)	47 (3.55)	0.200
LABA/ICS	407 (27.37)	2074 (29.25)	0.146	374 (28.23)	350 (26.42)	0.295
LAMA	225 (15.13)	1,102 (15.54)	0.690	214 (16.15)	167 (12.60)	0.009
LABA/LAMA	36 (2.42)	113 (1.59)	0.026	33 (2.49)	18 (1.36)	0.034
SABA	329 (22.13)	1982 (27.95)	<0.001	308 (23.25)	345 (26.04)	0.095
SAMA	146 (9.82)	1,050 (14.81)	<0.001	138 (10.42)	193 (14.57)	0.001
SABA/SAMA	243 (16.34)	1,351 (19.06)	0.015	231 (17.43)	234 (17.66)	0.878
Systemic beta-2 agonists	463 (31.14)	2,668 (37.63)	<0.001	419 (31.62)	487 (36.75)	0.005
Inhaled corticosteroid	76 (5.11)	407 (5.74)	0.338	67 (5.06)	82 (6.19)	0.206
Methylxanthine	854 (57.43)	4,657 (65.68)	<0.001	769 (58.04)	847 (63.92)	0.002
COPD severity						
Moderate exacerbations						
0	776 (52.19)	3,205 (45.20)	<0.001	667 (50.34)	681 (51.40)	0.817
1	257 (17.28)	1,106 (15.60)		212 (16.00)	202 (15.25)	
≥2	454 (30.53)	2,779 (39.20)		446 (33.66)	442 (33.36)	
Severe exacerbations						
0	921 (61.94)	3,722 (52.50)	<0.001	780 (58.87)	797 (60.15)	0.489
1	235 (15.80)	1,328 (18.73)		214 (16.15)	223 (16.83)	
≥2	331 (22.26)	2040 (28.77)		331 (24.98)	305 (23.02)	

COPD, chronic obstructive pulmonary disease; PH, pulmonary hypertension; TWD, Taiwan dollars; ASCVD, atherosclerotic cardiovascular disease; TIA, transient ischemic attack; CCB, calcium channel blocker; ACEI, angiotensin-converting enzyme inhibitor; ARB, angiotensin receptor blocker; LABA, long-acting β2-aginist; LAMA, long-acting muscarinic antagonists; SABA, short-acting β2-aginist; SAMA, short-acting muscarinic antagonists.

To identify similar patients in the control group, the study population was subjected to 1-to-1 propensity-score matching, and age group, sex, insurance premium, urbanization, hypertension, diabetes, HF, asthma, oxygen therapy, specific drug therapy and COPD severity were used to calculate the propensity score. Before matching, the propensity scores of the user and non-user groups were significantly different (0.77 ± 0.12 vs. 0.84 ± 0.10, *p* < 0.001). After matching, the propensity scores in the two groups were similar, with no significant differences (0.66 ± 0.04 vs. 0.66 ± 0.04, *p* = 1.000).

After matching, there were still significant differences in the distributions of comorbidity and co-medication between the two groups. The Poisson regression model and Cox proportional hazard model were used to adjust the imbalanced characteristics in the subsequent analysis. The detailed baseline characteristics of the PH cohort after 1-to-1 propensity-score matching are shown in Table 1.

### Mortality of Patients with Pulmonary Hypertension Related to Chronic Obstructive Pulmonary Disease

Regarding the primary outcome, during the five-year observation duration, 462 (34.87%) statin users and 647 (48.83%) statin non-users died, among whom 145 (10.94%) statin users and 210 (15.84%) statin non-users died of causes related to PH. Regarding the primary outcome, the total follow-up times of the user and non-user groups were 3,751.18 and 3,784.92 person-years, respectively. The statin user group had a lower mortality related to PH than the non-user group (3.87 vs. 5.55 per 100 person-years, *p* < 0.001). Regarding the secondary outcomes, most patients with mortality related to PH died due to RE (rates of mortality related to PH of the user and non-user groups: 86.21% and 79.52%), and few patients died due to PH or HF.

The Cox proportional hazard model analysis of primary outcome and secondary outcome of PH patients is summarized in Table 2. The univariate analysis (crude HR = 0.70, 95% CI = 0.56–0.86, *p* < 0.001) and multivariate analysis (aHR = 0.78, 95% CI = 0.62–0.98, *p* = 0.046) of mortality related to PH both showed significantly lower mortality for statin users than non-users. The above results suggested that statins could reduce the risk of mortality related to PH by approximately 22–30% and that they were effective in patients with PH related to COPD. Regarding secondary outcomes, there was significantly lower risk for deaths caused by PH or HF (aHR = 0.41, 95% CI = 0.23–0.74, *p* = 0.003) and all-cause mortality (aHR = 0.76, 95% CI = 0.67–0.87, *p* < 0.001).

**TABLE 2:** Cox proportional hazard model analysis of mortality of PH patients, stratified according to statin use.

	User n = 1,325			Non-user n = 1,325			Crude		Adjusted	
Outcomes	Event	Total of PY	Rate	Event	Total of PY	Rate	HR (95% CI)	*p*-value	HR^a^ (95% CI)	*p*-value
Primary outcome										
Mortality related to PH (PH, HF, RE)	145	3,751.18	3.87	210	3,784.92	5.55	0.70 (0.56–0.86)***	<0.001	0.78 (0.62–0.98)*	0.036
Secondary outcome										
All-cause mortality	463	3,751.18	12.34	647	3,784.92	17.09	0.72 (0.64–0.81)***	<0.001	0.76 (0.67–0.87)***	<0.001
Deaths caused by PH or HF	20	3,751.18	0.53	43	3,784.92	1.14	0.47 (0.28–0.80)**	0.005	0.41 (0.23–0.74)**	0.003
Deaths caused by RE	125	3,751.18	3.33	167	3,784.92	4.41	0.76 (0.60–0.95)*	0.017	0.90 (0.70–1.16)	0.401

^a^ Adjusted for age group, sex, income, comorbidity, co-medication, and COPD severity. HF, heart failure; PH, pulmonary hypertension; RE, respiratory exacerbation; HR, hazard ratio; Rate = (event/person-year) *100.

*<0.05; **<0.01; ***<0.001.

In the multivariate analysis, all baseline characteristics were included in the adjusted model. When statin users were over 70 years old, were male, had lower insurance premiums, had comorbidities including chronic kidney disease, interstitial pulmonary disease and HF, and were on oxygen therapy, they had a significantly higher risk of mortality than nonusers. Regarding COPD severity, patients with moderate exacerbation had a significantly higher risk than those with no moderate exacerbation. Furthermore, there was a trend in severe exacerbation in that patients with more severe exacerbations had a higher risk of PH (0, 1, and ≥2 times, aHR = 1.00, 1.25, and 2.55, respectively, *p* < 0.001). Table 3 showed multivariate Cox proportional hazard model analysis for variables related to all-cause mortality.

**TABLE 3:** Multivariate Cox proportional hazard model analysis for variables related to all-cause mortality.

Variables	Crude HR	(95% CI)	*p*-value	Adjusted HR^a^	(95% CI)	*p*-value
User vs. non-user	0.70	(0.56–0.86)**	0.001	0.78	(0.62–0.98)*	0.036
Age group^b^						
40 ≤ age <50 years	1	(Reference)		1	(Reference)	
50 ≤ age <60 years	2.14	(0.66–7.00)	0.208	1.94	(0.59–6.43)	0.276
60 ≤ age <70 years	2.24	(0.71–7.13)	0.171	2.54	(0.79–8.17)	0.118
70 ≤ age <80 years	2.80	(0.89–8.80)	0.078	2.70	(0.85–8.59)	0.092
Age ≥80 years	5.06	(1.61–15.88)**	0.006	5.07	(1.59–16.19)**	0.006
Male^b^	1.50	(1.21–1.87)***	<0.001	1.35	(1.07–1.70)*	0.011
Insurance premium (NT$)^b^						
≤22,800	1	(Reference)		1	(Reference)	
>22,800	0.56	(0.44–0.71)***	<0.001	0.71	(0.54–0.92)**	0.010
Urbanization level						
Urban	1	(Reference)		1	(Reference)	
Suburban	1.01	(0.81–1.26)	0.913	0.97	(0.78–1.22)	0.819
Rural	1.00	(0.71–1.40)	0.990	0.88	(0.62–1.24)	0.460
Comorbidity						
Hypertension	0.98	(0.78–1.23)	0.862	0.79	(0.60–1.03)	0.079
Diabetes mellitus	1.02	(0.82–1.26)	0.884	0.80	(0.60–1.06)	0.114
Chronic kidney disease^b^	1.42	(1.03–1.95)	0.033	1.13	(0.81–1.59)	0.470
Chronic liver disease	1.18	(0.84–1.65)	0.343	1.14	(0.81–1.61)	0.457
Arrhythmia	1.13	(0.90–1.41)	0.290	0.89	(0.69–1.14)	0.347
Interstitial pulmonary disease^b^	3.32	(2.11–5.23)***	<0.001	3.02	(1.89–4.84)***	<0.001
Asthma	1.11	(0.89–1.38)	0.352	0.94	(0.74–1.18)	0.567
Malignancy	0.94	(0.67–1.32)	0.719	0.80	(0.56–1.13)	0.201
ASCVD						
Coronary artery disease	1.04	(0.84–1.28)	0.710	0.97	(0.77–1.22)	0.795
Peripheral vascular disease	1.12	(0.71–1.75)	0.635	1.09	(0.69–1.73)	0.709
Ischemic stroke/TIA	0.94	(0.69–1.28)	0.694	0.77	(0.56–1.06)	0.104
Hemorrhagic stroke	1.87	(0.88–3.95)	0.102	1.29	(0.60–2.78)	0.515
Heart failure^b^	1.86	(1.50–2.31)***	<0.001	1.47	(1.14–1.90)**	0.003
Left ventricular hypertrophy	0.87	(0.45–1.69)	0.683	0.81	(0.41–1.59)	0.540
Co-medication						
Digoxin	1.43	(1.13–1.82)**	0.003	1.18	(0.90–1.54)	0.232
Oral anticoagulant agents	1.07	(0.78–1.47)	0.675	1.14	(0.81–1.62)	0.450
Oral antiplatelet agents	0.97	(0.78–1.19)	0.740	1.06	(0.83–1.36)	0.637
Diuretics	1.55	(1.23–1.96)***	<0.001	1.08	(0.83–1.40)	0.572
CCB	0.95	(0.77–1.17)	0.620	0.96	(0.77–1.21)	0.754
ACEI/ACB	0.93	(0.75–1.15)	0.499	0.91	(0.71–1.16)	0.433
Beta blocker	0.61	(0.48–0.78)***	<0.001	0.69	(0.53–0.90)**	0.005
Metformin	0.84	(0.64–1.10)	0.209	0.96	(0.68–1.36)	0.821
Fibrates	0.25	(0.09–0.66)**	0.005	0.36	(0.14–0.99)*	0.047
Other lipid-lowering drugs	0.65	(0.09–4.58)	0.661	0.68	(0.09–4.93)	0.699
Oxygen therapy^b^	3.30	(2.61–4.17)***	<0.001	1.77	(1.34–2.33)***	<0.001
Specific drug therapy	1.01	(0.25–4.04)	0.994	0.85	(0.21–3.55)	0.828
COPD severity						
Moderate exacerbations^b^						
0	1	(Reference)		1	(Reference)	
1	2.40	(1.73–3.32)***	<0.001	1.89	(1.35–2.66)***	<0.001
≥2	3.12	(2.40–4.05)***	<0.001	1.48	(1.08–2.04)*	0.016
Severe exacerbations^b^						
0	1	(Reference)		1	(Reference)	
1	1.62	(1.16–2.26)**	0.005	1.25	(0.89–1.74)	0.197
≥2	4.27	(3.36–5.42)***	<0.001	2.55	(1.87–3.48)***	<0.001

^a^Adjusted for age group, sex, income, comorbidity, co-medication, and COPD severity.

b Entry regression model after the stepwise multiple regression analysis. COPD, chronic obstructive pulmonary disease; PH, pulmonary hypertension; HR, hazard ratio; TWD, Taiwan dollars; ASCVD, atherosclerotic cardiovascular disease; TIA, transient ischemic attack; CCB, calcium channel blocker; ACEI, angiotensin-converting enzyme inhibitor; ARB, angiotensin receptor blocker.

*<0.05; **<0.01; ***<0.001.

Stepwise multiple regression analysis was then conducted to identify the main factors affecting the mortality rate of patient with PH. Only age group, sex, insurance premium, chronic kidney disease, interstitial pulmonary disease, HF, oxygen therapy and COPD severity were significant risk factors. Those significant factors were used to construct the adjusted model for the subsequent subgroup analysis.

### Subgroup Analysis

#### Different Kinds of Statins

The most commonly used statin was atorvastatin, with 579 (44%) statin users taking this statin. The least commonly used statin was pitavastatin. The Cox proportional hazard model analysis of all-cause mortality in different kinds of statins is summarized in Table 4. The reference for each statin was the matched patients in the statin non-user group.

**TABLE 4:** Subgroup analysis of the risk of mortality-related PH for different statins stratified according to statin use.

Statins	N	User n = 1,325			Non-user n = 1,325			Crude HR	(95% CI)	*p*-value	Adjusted HR^a^	(95% CI)	*p*-value
		Events	Total of PY	Rate	Events	Total of PY	Rate						
Simvastatin	157	15	485.55	3.09	24	506.08	4.74	0.65	(0.34–1.24)	0.188	0.65	(0.34–1.26)	0.200
Lovastatin	79	6	265.41	2.26	14	213.65	6.55	0.35	(0.13–0.91)*	0.031	0.36	(0.14–0.97)	0.044
Pravastatin	76	11	186.18	5.91	13	187.21	6.94	0.84	(0.38–1.88)	0.677	0.76	(0.33–1.75)	0.516
Fluvastatin	101	11	325.18	3.38	18	304.03	5.92	0.57	(0.27–1.20)	0.137	0.58	(0.27–1.25)	0.165
Atorvastatin	579	74	1,604.59	4.61	81	1,634.74	4.95	0.93	(0.68–1.28)	0.661	1.00	(0.73–1.38)	0.994
Rosuvastatin	286	27	783.93	3.44	55	789.27	6.97	0.49	(0.31–0.78)**	0.003	0.51	(0.31–0.82)	0.005
Pitavastatin	47	1	100.34	1.00	5	149.94	3.33	0.36	(0.04–3.07)	0.347	0.32	(0.03–3.25)	0.335

^a^ Adjusted for age group, sex, insurance premium, chronic kidney disease, interstitial pulmonary disease, heart failure, oxygen therapy, and COPD severity.

HR, hazard ratio; PH, pulmonary hypertension; PY, person-year; Rate = (event/person-year) *100.

*<0.05; **<0.01; ***<0.001.

All of the statins showed a trend toward low risk of mortality (crude HR < 1.00). Patients using lovastatin (aHR = 0.36, 95% CI = 0.14–0.97, *p* = 0.044) and rosuvastatin (aHR = 0.51, 95% CI = 0.31–0.82, *p* = 0.005) had a significantly lower risk of mortality. Among all statins, lovastatin had the lowest mortality rate (aHR = 0.36, 95% CI = 0.14–0.97, *p* = 0.044).

#### Cumulative Defined Daily Doses

The cDDD was calculated as total statin exposure standardized by DDD over the whole follow-up period. There were six levels of classification for cDDD, and a multivariate Cox proportional hazard model analysis was performed. The statin non-user group was a reference in the analysis. Forty percent of statin users had a cDDD of more than 730. Table 5 shows that the mortality risk of the statin user group was significantly lower than that of the nonuser group until the cDDD was over 365 (aHR = 0.60, 95% CI = 0.40–0.90, *p* = 0.013). Additionally, patients with a cDDD above 365 had dose-dependent response. The higher the cDDD was, the lower the risk of mortality would be (HR from 1.21 to 0.45, *p* trend=<0.001).

**TABLE 5:** Subgroup analysis of the risk of PH-related mortality, stratified according to the classification of cDDD, duration of statin use, and intensity.

Group	N	Events	Total of PY	Rate	Crude HR	(95% CI)	*p*-value	Adjusted HR^a^	(95% CI)	*p*-value
cDDD										
Non-user	1,325	210	3,784.92	5.55	1	(Reference)		1	(Reference)	
cDDD <28	78	15	214.53	6.99	1.26	(0.75–2.13)	0.390	1.21	(0.72–2.05)	0.473
28 ≤ cDDD <90	120	20	343.18	5.83	1.05	(0.67–1.67)	0.827	1.02	(0.65–1.62)	0.924
90 ≤ cDDD <180	130	24	314.05	7.64	1.37	(0.90–2.10)	0.140	1.27	(0.83–1.94)	0.270
180 ≤ cDDD <365	216	28	631.77	4.43	0.80	(0.54–1.19)	0.264	0.77	(0.52–1.15)	0.198
365 ≤ cDDD <730	292	26	837.51	3.10	0.56	(0.37–0.84)**	0.005	0.60	(0.40–0.90)*	0.013
cDDD ≥730	489	32	1,410.14	2.27	0.41	(0.28–0.59)***	<0.001	0.45	(0.31–0.66)***	<0.001
*p* trend	<0.001									
Duration of statin use (years)										
Non-user	1,325	210	3,784.92	5.55	1	(Reference)		1	(Reference)	
<0.5 years	211	41	594.19	6.90	1.24	(0.89–1.74)	0.202	1.17	(0.84–1.64)	0.348
≥0.5 years but <1 year	153	22	418.07	5.26	0.95	(0.61–1.47)	0.813	0.99	(0.64–1.54)	0.960
≥1 year but <2 years	223	19	616.82	3.08	0.55	(0.35–0.89)*	0.014	0.56	(0.35–0.89)*	0.015
≥2 years but <3 years	188	18	557.50	3.23	0.58	(0.36–0.94)*	0.027	0.60	(0.37–0.97)*	0.037
≥3 years	550	45	1,564.59	2.88	0.52	(0.38–0.72)***	<0.001	0.57	(0.41–0.78)**	0.001
*p* Trend	<0.001									
Intensity (cDDD/month)										
Non-user	1,325	210	3,784.92	5.55	1	(Reference)		1	(Reference)	
Intensity <10	131	27	310.64	8.69	1.56	(1.05–2.33)*	0.029	1.46	(0.97–2.18)	0.068
Intensity≥ 10 but <20	686	71	2023.25	3.51	0.63	(0.48–0.83)**	0.001	0.64	(0.49–0.84)**	0.001
Intensity ≥20	508	47	1,417.29	3.32	0.60	(0.44–0.82)***	<0.001	0.66	(0.48–0.91)*	0.010
*p* trend	<0.001									

a Adjusted for age group, sex, insurance premium, chronic kidney disease, interstitial pulmonary disease, heart failure, oxygen therapy, and COPD severity.

COPD, chronic obstructive pulmonary disease; PH, pulmonary hypertension; cDDD, cumulative defined daily doses; PY, person-year; Rate = (event/person-year) *100; HR, hazard ratio; *<0.05; **<0.01; ***<0.001.

### Duration of Statin Use (years)

The duration of statin use was calculated by year and divided into six levels. As shown in Table 5, multivariate Cox proportional hazard model analysis revealed that patients who had a longer duration of statin use had a lower risk of mortality. When patients used statins for longer than 1 year, they started to have a significantly lower risk of mortality (aHR = 0.56, 95% CI = 0.35–0.89, *p* = 0.015). This result suggested that statin use was effective against PH when the duration of use was longer than one year.

### Intensity of Statin Use (Cumulative Defined Daily Doses/Month)

The intensity of statins was calculated by dividing cDDD by the duration of statin use over the entire five-year observation period. Then, the patients were divided into four levels, and multivariate Cox proportional hazard model analysis was performed. The normal DDD per month of statins is 30. Table 5 shows that patients who had a higher statin DDD per month had a lower risk of mortality (aHR = 1.46 to 0.66, *p* trend=<0.001). Patients whose statin DDD was above 10 per month started to have a significantly lower risk of mortality (aHR = 0.64, 95% CI = 0.49–0.84, *p* = 0.001). This result suggested that statins had a protective effect against PH until the patients the DDD exceeded 10 per month. Table 5. Subgroup analysis of the risk of PH-related mortality, stratified according to the classification of cDDD, duration of statin use, and intensity.

### Sensitivity Analysis

#### Different Observation Durations

As presented in Table 6, except for that of the one-year observation duration, the aHR of each observation period was similar to the aHR originally observed for five years. After the multivariate Cox proportional hazard model analysis was performed, the 3-year, 5-year, 7-year, 9-year, and end-of-study timepoints still had significantly lower aHRs than the statin non-users. Table 6 showed Sensitivity analysis of risk of mortality during different follow-up times.

**TABLE 6:** Sensitivity analysis of risk of mortality during different follow-up times, stratified according to statin use.

Follow-up period	User (n = 1,325)			Non-user (n = 1,325)			Crude HR	(95% CI)	*p*-value	Adjusted HR^a^	(95% CI)	*p*-value
	Events	Total of PY	Rate	Event	Total of PY	Rate						
1 year	53	1,170.11	4.53	56	1168.97	4.79	0.95	(0.65–1.38)	0.770	1.08	(0.72–1.63)	0.715
3 years	110	2,778.99	3.96	152	2,794.41	5.44	0.73	(0.57–0.93)**	0.011	0.82	(0.63–1.07)	0.151
5 years	145	3,751.18	3.87	210	3,784.92	5.55	0.70	(0.56–0.86)***	<0.001	0.78	(0.62–0.98)*	0.036
7 years	167	4,321.26	3.86	237	4,362.17	5.43	0.71	(0.58–0.87)***	<0.001	0.79	(0.64–0.98)*	0.030
9 years	178	4,602.31	3.87	257	4,707.42	5.46	0.71	(0.58–0.86)***	<0.001	0.79	(0.64–0.97)*	0.023
End of study	185	4,769.92	3.88	269	4,925.81	5.46	0.71	(0.59–0.86)***	<0.001	0.80	(0.65–0.98)*	0.032

^a^ Adjusted for age group, sex, income, comorbidity, co-medication, and COPD severity. PH, pulmonary hypertension; ratio; PY, person-year; Rate = (event/person-year) *100; HR, hazard ratio; *<0.05; **<0.01; ***<0.001.

## Discussion

In our study, statins were effective in improving PH outcomes in COPD. Regarding the primary outcome, the five-year cumulative mortality was 10.94% for statin users and 15.84% for statin non-users. The PH patients with the statin user group had a lower mortality rate than those in than the non-user group (3.87 vs. 5.55 per 100 person-years, *p* < 0.001) After adjustments for risk factors in the Cox proportional hazard model analysis, the results maintained a significantly lower risk of PH mortality. These results suggest that statins may decrease the risk of mortality from PH by 22% and show a benefit for PH patients.

### Reverse Causality

To resolve the reverse causality, we set a six-month drug washout period according to the pharmacokinetic and pharmacodynamic properties of statins (Liu et al., 2016a; Liu et al., 2016b). The half-lives of statins are between 1.2–19 h. It is generally believed that four to five half-lives (i.e., 1–5 days for statins) are needed to excrete most of the drugs from the body (Schachter, 2005). A study involving a mouse model showed that in smooth muscle cells and vascular endothelial cells, discontinuation of atorvastatin (half-life: 14 h) for 2 days caused a 90% decrease in nitric oxide (NO) production. (Laufs et al., 2000). Another study of 21 patients showed reduced endothelial NO synthase activity and NO levels after withdrawal of fluvastatin (half-life: 1.2 h) for 24 h (Westphal et al., 2008; Pineda and Cubeddu, 2011).

According to the above, we think that the six-month washout period was reasonable and long enough to ensure that statins did not influence the COPD diagnosis. Therefore, patients with any statin exposure during the washout period were excluded.

### Chronic Obstructive Pulmonary Disease Severity

Because of the lack of forced expiratory volume in one second (FEV1) and exam results in the NHI database, it was difficult to confirm COPD severity in patients. We used the assessment of exacerbation risk in the Global Initiative for Chronic Obstructive Lung disease guidelines to define the severity of COPD. The assessment tool is divided into high-risk and low-risk groups according to the number of exacerbations over the previous year. Severe exacerbations are exacerbations leading to emergency room or hospital admission, and moderate exacerbations are exacerbations were treated with SABA plus antibiotics or oral corticosteroids. If patients had ≥2 moderate exacerbations or ≥1 severe exacerbation over the previous year, they were considered have a high exacerbation risk. If patients had no exacerbations or one moderate exacerbation, they were considered to have a low exacerbation risk (Sun and Ku, 2008). The severity of COPD was confirmed one year after COPD diagnosis.

### Pathogenesis of Pulmonary Hypertension in Chronic Obstructive Pulmonary Disease

Because of the pathogenesis of PH involves proliferation of lung vasculature, vasoconstriction, and thrombosis, the anti-proliferative effects and antithrombotic effects of statins as a RhoA/Rho-kinase inhibitions seem to be benefic on PH (Seeger et al., 2000; Sun and Ku, 2008).

Although statins improve systemic inflammatory, pulmonary vascular proliferation, and block the RhoA/Rho-kinase signaling pathway *in vitro* studies, the efficacy of statins in clinical trials is still unclear. Each statin seems to have a different contribution to protective effect and effectiveness. Compare to pharmacokinetic and pharmacodynamics property of each statin, they were no consistent in bioavailability, protein binding, lipophilicity, half-life, and other metabolism properties. Besides, we could not find studies about comparing the effects of anti-proliferative and anti-inflammatory or the potency of RhoA/Rho-kinase inhibition. Many randomized controlled trials support the benefit of statins in patients with PH related to COPD. In 2009, Lee et al. performed the first randomized controlled trial to explore the effectiveness of statins in this population. After using pravastatin 40 mg one per day for six months, the statin users showed significantly improved exercise tolerance and decreased PH and dyspnea during exercise compared to the placebo group (*p* < 0.001). Recent studies of simvastatin, rosuvastatin, and atorvastatin showed that they consistently and significantly improved 6 min walking distance results and directly reduced pulmonary artery pressure (Shahmiri et al., 2009; Chogtu et al., 2016; Arian et al., 2017). According to the comprehensive prognostic evaluation and risk assessment in PH, exercise tolerance and pulmonary artery pressures can predict one-year mortality. Therefore, we can presume that if statins can improve these assessment items, statins may be effective for reducing the mortality of PH.

Regarding the secondary outcome, the rates of all-cause mortality (12.34 vs. 17.09 per 100 person-years, *p* < 0.001) and deaths caused by PH or HF (0.53 vs. 1.14 per 100 person-years, *p* = 0.005) were a significantly lower in the statin user group than in the non-user group. Although most patients died due to RE, the findings was only a trend of lower risk, with no significant difference. The distribution of causes of death was similar to the most common symptoms that caused patient hospitalization in the Chen et al. study. The majority of patients were hospitalized for dyspnea (93.5%), cough (89.8%), and sputum (79.7%) rather than symptoms caused by PH or HF, such as edema (8.2%) (Chen et al., 2016).

### Statin and Prognosis

In previous randomized controlled trials, although statins showed some benefits for PH, the assessments of lung function and heart function, including cardiac output, right ventricular size, FEV1, and lung capacity, did not show significant differences compared to the placebo groups. Only the findings of Chogtu et al.’s study, which showed a significant reduction in COPD exacerbations, are consistent with those of this study (Chogtu et al., 2016). Death can have different causes, so it is difficult to distinguish which statin has the greatest efficacy in reducing the incidence of a specific cause of death. The secondary outcomes only suggest that statins may have an association with a lower risk of RE-related mortality.

In our study, the most commonly used statin was atorvastatin, with nearly 40% of users taking this statin, and the least used statin was pitavastatin, because pitavastatin was the most recent statin to be included in the National health insurance program. Most statins had a trend toward reducing the incidence of PH incidence and the risk of mortality; however, patients in part I who used only pravastatin and atorvastatin had a significantly lower incidence, and patients in part II using lovastatin and rosuvastatin had a significantly lower risk of mortality. The results revealed that the statins seem to have a differing levels of protection and efficacy. When the pharmacokinetic and pharmacodynamic properties of each statin are compared, it becomes apparent that the statins are not the same in terms of bioavailability, protein binding, lipophilicity, half-life, or other metabolic properties. In addition, we could not find studies comparing the antiproliferative and anti-inflammatory effects or the potency of RhoA/Rho-kinase inhibition by these statins.

The probable reason for the inconsistent statin effect between incidence and mortality is that some patients switched to another statin during the observation duration. Therefore, it was difficult to distinguish which statins contributed to the effect. Another reason is the definition of statin grouping. The study divided patients into each statin group according to the statin with the highest cDDD during the observation duration. Perhaps the first statin that the patient was exposed to after the index date or the most recent statin treatment relative to the events of the patient have more influence on the effect. Until now, randomized controlled trials have been performed for only pravastatin, simvastatin, atorvastatin, and rosuvastatin to explore their effects on PH. More direct or indirect comparative studies of statins are needed to support these results.

### Dose-dependent Effects

In the subgroup analysis, the results of two parts showed a dose-dependent effect and time-dependent effect. When patients had a higher cDDD and longer duration of statin use, they had a lower risk of PH and mortality related to PH.

To understand the association between cDDD and the duration of statin use, a subgroup analysis of intensity was performed. The intensity indicates the cDDD of statins per month. The normal cDDD per month is 30. However, the DDD is an average maintenance dose per day, so it may represent a moderate intensity of statin use according to their lipid-lowering effects (Wierzbicki et al., 2003; Grundy et al., 2018). Patients with a statin DDD above 20 per month started to have a significantly lower risk of PH (aHR = 0.54, 95% CI = 0.41–0.71), and those with a DDD above 10 per month started to have a significantly lower risk of mortality related to PH (aHR = 0.64, 95% CI = 0.49–0.84, *p* = 0.001).

The dose-dependent and time-dependent effects of statins are common to many studies in different populations, making the observed effect more robust (Liu et al., 2016a; Liu et al., 2016b; Langballe et al., 2018). In summary, the results suggest that the statins have a protective effect against PH and are effective against PH when patients use them for more than 1 year and at a DDD of more than 10 to 20 per month.

### Strengths and Limitations

To our knowledge, this study is the first to explore the protective effect of statins against PH in COPD patients. Moreover, it is the first population-based cohort study to explore the protective effect and efficacy of statins in COPD patients. There are two strengths in our study. First, the NHI database is one of the largest and most comprehensive medical population databases in the world. The data can be extracted from 1995 to 2017 and cover 99.6% of the 23 million people in the Taiwan population. The study had a large sample size, even after propensity-score matching was conducted. There were 41,163 COPD patients and 1,325 patients with PH related to COPD in the final analysis of the study. In contrast to that of other studies, the sample size of patients with PH related to COPD is only 40 to 60 patients (Lee et al., 2009; Wilkins et al., 2010; Zeng et al., 2012; Moosavi et al., 2013; Chogtu et al., 2016; Arian et al., 2017). In general, the larger sample size may provide enough power for statistical analysis. Another strength is the observation period. The study used the database from 2002 to 2017 and set a five-year observation duration. The long observation period is very important in the study. Our study could evaluate the long-term mortality of PH patients, in contrast to other RCT studies, which had follow-up periods of only six months or less. To confirm that the observation time was appropriate, different observation times were tested in the sensitivity analysis. Moreover, the study used event-driven endpoints including the morbidity and mortality of PH.

In addition, RCTs include only more stable PH patients and relatively healthier patients. Chogtu et al. included patients with COPD who were stable for at least 2 weeks and excluded patients with asthma and cardiac disorders such as arrhythmias and unstable angina (Arian et al., 2017). Our study assessed whether COPD severity influences the protective effect and efficacy of statins. Furthermore, the results were robust across several different definitions of statin drug exposure and observation durations. However, we could not find a consistent benefit in terms of protective effect and efficacy between different kinds of statins. Further studies with different statins and accurate statin exposure control may be required.

This study has several limitations. The disease diagnosis and severity assessments of the morbid conditions may not accurately reflect the real situation in this study that could have influenced the outcome of the measurements. The absence of information relating to other factors such as cigarette smoking cannot be underestimated. The study population and outcomes were all defined based on the International Classification of Diseases, Ninth Revision, Clinical Modification or International Classification of Diseases, Ten Revision, Clinical Modification in the admissions record and not on clinical diagnosis because the National health insurance database lacks examination results and lab data. Although we used multiple definitions, such as combined treatment or examination, to improve accuracy, uncertain error is still expected. The limitation of the National health insurance database also leads to the study not having access to smoking status, lifestyle, lung function, or PH severity. Therefore, adjustments could not be made for these important factors or potential confounders, which may influence the outcomes.

## Conclusion

Our study suggests that statins have protective effects and efficacy against PH related to COPD and can reduce the mortality of PH. Moreover, the benefits of statins are dose dependent and time dependent.

Age over 60 years, male, low income, HF, and COPD severity are risk factors for mortality, and chronic kidney disease and interstitial pulmonary disease are risk factors for mortality among PH patients.

## Data Availability

Data are available from the National Health Insurance Research Database (NHIRD) published by the Taiwan National Health Insurance (NHI) Bureau. Due to legal restrictions imposed by the government of Taiwan in relation to the “Personal Information Protection Act,” data cannot be made publicly available. Requests to access the datasets should be directed to, jk2975525@hotmail.com.

## Abbreviations:

ACEI, angiotensin-converting enzyme inhibitor; ARB, angiotensin receptor blocker; ASCVD, atherosclerotic cardiovascular disease; CCB, calcium channel blocker; cDDD, cumulative defined daily doses; COPD, chronic obstructive pulmonary disease; HF, heart failure; HR, hazard ratio; LABA, long-acting β2-aginist; LAMA, long-acting muscarinic antagonists; PH, pulmonary hypertension; PY, person-year; SABA, short-acting β2-aginist; SAMA, short-acting muscarinic antagonists; TIA, transient ischemic attack; TWD, Taiwan Dollars.
